# Renal hydatid cyst: a rare case

**DOI:** 10.11604/pamj.2024.48.175.44470

**Published:** 2024-08-14

**Authors:** Ashwin Karnan

**Affiliations:** 1 Department of Respiratory Medicine, Jawaharlal Nehru Medical College, Datta Meghe Institute of Higher Education and Research, Sawangi (Meghe), Wardha, Maharashtra, India

**Keywords:** Echinococcus, dysuria, kidney, cystitis

## Image in medicine

A 32-year-old male, farmer, presented with chief complaints of fever, right-sided flank pain, and burning micturition for the past 3 weeks. The patient gives a history of having pet dogs at home. Computed Tomography (CT) urogram showed a well-defined multiseptated calcified cystic lesion of size 9 x 7 cm involving the right kidney's upper, mid, and lower pole. A diagnosis of urinary tract infection or complex kidney cyst was considered. Urine examination showed hydatid cysts, Serum IgG for *Echinococcus* and Casoni test was positive. Pre-operatively the patient was treated with the tablet Albendazole and with the help of a general surgeon and urosurgeon, the patient underwent laparoscopic cystectomy. The patient improved symptomatically and is currently on follow-up. *Echinococcosis* is a zoonotic disease commonly caused by *E. granulosus*. The liver and lungs are the most common sites followed by the brain, bones, kidney, and pancreas. Renal hydatid disease accounts for about 2% of hydatid diseases. It more commonly affects children than adults. Ultrasonogram is the mainstay of diagnosis showing cystic mass with daughter cysts. Other diagnostic modalities include the Casoni test, Ghedine-Weinberg test, and serum IgG antibodies for *Echinococcus*. Medical management of renal hydatid disease is not possible. Treatment options include percutaneous repair, minimally invasive surgery or open surgery. Surgical management involves cystectomy or pericystectomy and in cases where renal parenchyma is involved, partial or complete nephrectomy may be done.

**Figure 1:**
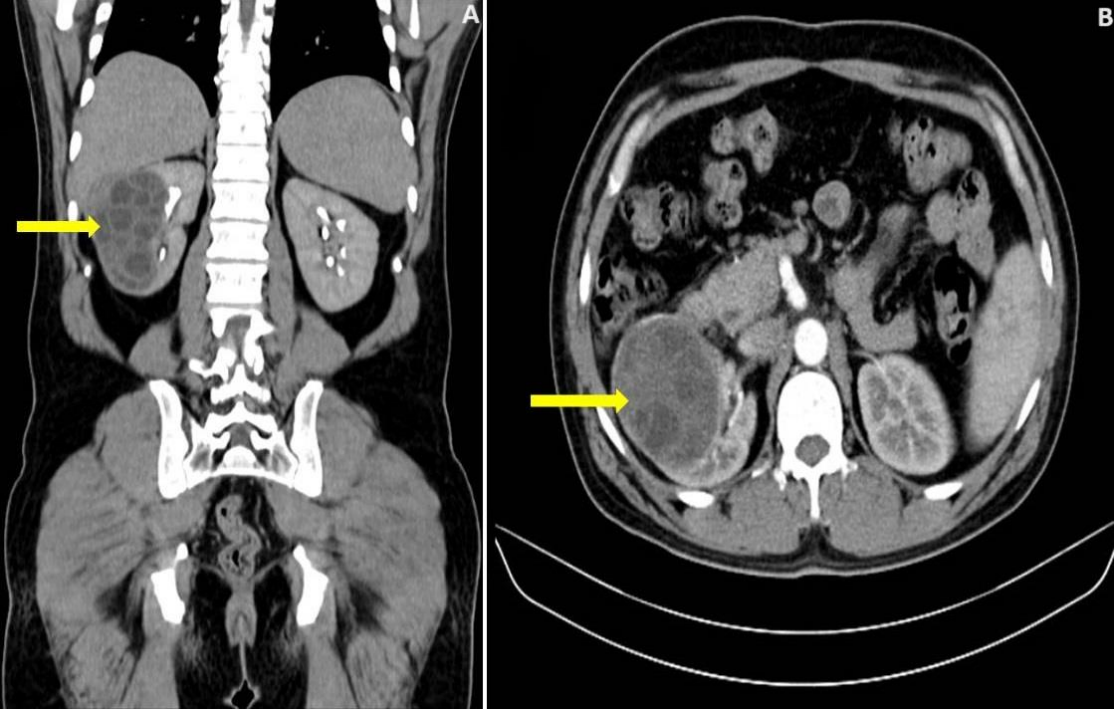
(A,B) CT scan of the abdomen with a yellow arrow showing multiloculated cystic lesion with calcifications in the right kidney

